# Protocol for a web survey experiment studying the feasibility of asking respondents to capture and submit photos of the books they have at home and the resulting data quality

**DOI:** 10.12688/openreseurope.16507.1

**Published:** 2023-11-20

**Authors:** Patricia A. Iglesias, Melanie Revilla, Birgit Heppt, Anna Volodina, Clemens Lechner

**Affiliations:** 1 Research and Expertise Centre for Survey Methodology, Department of Political and Social Sciences, Universitat Pompeu Fabra, Barcelona, Catalonia, 08005, Spain; 2 Institut Barcelona d'Estudis Internacionals, Barcelona, Catalonia, 08005, Spain; 3 Humboldt-Universitat zu Berlin, Berlin, Berlin, Germany; 4 Institute for Educational Quality Improvement at the Humboldt-Universitat zu Berlin, Berlin, Berlin, Germany; 5 GESIS – Leibniz Institute for the Social Sciences, Mannheim, Germany

**Keywords:** images, image classification, visual data capture, data quality, mobile web surveys, books at home.

## Abstract

This document presents the protocol of a study conducted as a part of the WEB DATA OPP project, which is funded by the H2020 program. The study aimed to investigate different aspects of the collection of images through web surveys. To do this, we implemented a mobile web survey in an opt-in online panel in Spain. The survey had various questions, some of which were about the books that the participants have at their main residence. The questions related to books were asked in three different ways: regular survey questions showing visual examples of how different numbers of books fit in a 74 centimetre wide shelf depending on their thickness, regular survey questions without the visual examples, and questions where participants were asked to send photos of the books at their home. This report explains how the study was designed and conducted. It covers important aspects such as the experimental design, the questionnaire used, the characteristics of the participants, ethical considerations, and plans for disseminating the results.

## Plain language summary

This document presents the protocol of our study asking respondents for information about the books they have at home. This information was solicited through conventional types of questions ( *i.e.,* typing in answers or choosing one answer category), and/or through asking respondents to take and send photos of the books.

This study has methodological and substantive objectives. The former involves investigating respondents’ preference, evaluation of the questions, participation levels, compliance, and data quality. The latter focuses on exploring the impact of the number of books on the academic achievement of children and examining other factors that might influence these relations.

We conducted a mobile web survey, assigning respondents to four groups:

•   Choice: Respondents could choose their preferred answering method.

•   Text-TextPlus: Respondents answered conventional questions first, and later received illustrations of how different numbers of books looked like to help respondents provide accurate answers.

•   TextPlus-Images: Respondents answered conventional questions with the illustrations and then submitted photos of the books at home.

•   Images-Text: Respondents shared photos of the books and then answered the conventional questions.

Respondents were asked to evaluate their respective answering methods. The questionnaire had up to 65 questions covering various dimensions, including respondents’ sociodemographic characteristics, children's academic performance, literacy-related activities, and camera usage.

Data were collected using the Netquest opt-in online panel in Spain. The tool *WebdataVisual* was used to capture and share the photos. The target population included parents of children living with them and who attended the first, third, or fifth year of primary school. The sample size was 1,202 cases.

We expect this study to provide valuable insights regarding visual data collection through web surveys. Further, we expect to gain a better understanding of the data on the books respondents have at home when such data are collected through different methods.

## Project overview

This study is part of the project WEB DATA OPP, which investigates how new measurement opportunities linked to the growing presence of mobile devices can help scientists and practitioners to get more accurate and/or new insights than using conventional web surveys. While four types of new data and collection techniques are being studied in this project, the current study focuses on the collection of visual data, particularly photos taken by the respondents during a web survey with their smartphone or tablet.

## Protocol study status

Data was collected in June 2023 and some preliminary analyses (regarding participation) have already taken place. Images have been reviewed by the fieldwork company (Netquest) and the project’s Ethics Advisor, and have been received by the research team, but they have not been reviewed yet. Manual classification of the images is scheduled to start in September 2023.

## Introduction

It is very common in social-science surveys to ask about the number of books respondents have at home (see, for example, Brunello *et al.*, 2012; Engzell, 2021; McNally *et al.*, 2023; Sanders *et al.*, 2004) as a proxy to measure the levels of cultural capital ( Sieben & Lechner, 2019) and/or socioeconomic capital of a certain person or group ( Asadullah & Tham, 2023; Heppt *et al.*, 2022). Typically, such questions are asked along with multiple other questions that allow characterizing the cultural and/or the socioeconomic capital of the subject(s), such as the presence and/or frequency of cultural activities, income, and educational level.

However, the most commonly used types of question about the number of books present several limitations. First, people usually do not know the number of books in their home. At best, they might estimate the approximate number based on their own calculations. The accuracy of this estimate is subject to variability based on various factors, including the mathematical capabilities of the respondents, their living situations ( *e.g.,* whether they live alone or with others), and the accessibility of their books.

Second, social desirability bias ( Edwards, 1957) can be expected because having many books could be perceived as a positive characteristic that signals social status. Thus, respondents might tend to over-report the number of books they have.

Third, the question on the number of books is often asked with an answer scale proposing intervals so that respondents might choose the one that best fits their situation. For instance, the response scale proposed by Bezek Güre *et al.* (2023) is: 0–10 book(s), 11–25 books, 26–100 books, 101–200 books, 201–500 books, and more than 500 books. The width of such intervals (especially the last three) does not allow to know, or even approximate, the exact number of books. Furthermore, the intervals are arbitrary, and they might influence respondents’ answers by suggesting what is a low or high level of books.

In addition, if researchers are interested in measuring cultural capital, this question might not provide sufficiently detailed information. Specifically, as cultural capital is a complex construct, it might be important to not only consider the mere number of books, but also their content (for a comprehensive discussion of cultural capital, see Bourdieu, 1986). For instance, are 30 cooking books related to the same level of cultural capital than 30 history books? Thus, it might be relevant to consider the type of books, or other aspects ( *e.g.,* language) to better measure cultural capital. This information can be obtained through additional survey questions, but at the cost of an additional burden to respondents.

To overcome these limitations, in this study, we conduct a mobile web survey experiment to assess the feasibility of asking respondents to capture and submit photos of the books they have at home and the resulting data quality. We expect that asking for images (compared to asking a conventional survey question) will: a) produce higher non-response, but b) provide more objective and accurate estimates of the actual number of books, as well as c) allow for collecting additional information regarding the books’ characteristics.

Empirical evidence so far regarding the participation when collecting images in the frame of web surveys, and specifically photos captured within a survey, is scarce and varies considerably. The participation rates range from 10% in the study by Jäckle *et al.* (2019) asking for photos of receipts, to 55% in the study by Bosch *et al.* (2019a) when asking for a photo of the place where respondents are when answering the survey. Moreover, greater item nonresponse has been found when respondents had to answer by sharing images than when using conventional response formats ( Bosch *et al.*, 2022; Ilic *et al.*, 2022).

As for the respondents’ evaluation with surveys asking for images, the literature is also scarce. Bosch *et al.* (2022) found that between 12.4% and 20.0% of respondents liked answering with images, while 47.3% to 52.7% liked answering similar questions in conventional ways. Further, nearly 80% of respondents found it easy to answer in conventional ways, versus around 50% for answering by sharing images, which also resulted taking more time for participants. However, in a study where respondents could only answer by sharing images, the assessment of easiness presented better results: 82.1% of respondents found it somewhat or very easy to complete the study ( Read, 2019). The scarcity and significant disparity of empirical evidence makes further research imperative to obtain more conclusive results.

Regarding preferences, to the best of our knowledge, there is only one survey proposing participants to choose in advance between only images or only conventional ways of answering ( Ilic *et al.*, 2022): they found that more than half (57%) of their sample chose images.

Even less empirical evidence exists about the quality of the data when it comes to the collection of images through web surveys. The few existing studies have mostly focused on whether the images contained information in line with what was asked. They have found that between 12.5% ( Ilic *et al.*, 2022) and 22.8% ( Bosch *et al.*, 2019a) of the images submitted were off topic. However, research delving into other indicators of quality for image collection through web survey is still needed.

Further, images have been used for substantive analyses although not always within the frame of web surveys. For instance, they have been collected to identify mosquitoes, with photos sent by voluntary participants who use the Mosquito Alert app ( Pataki *et al.*, 2021), or to assess plant diseases (see Kaur *et al.*, 2019). When it comes to substantive analyses asking for images within the frame of surveys, the most remarkable example was the @HBS project (HBS stands for Household Budget Survey), which tested the use of an app to collect photos of receipts for a Household Budget Survey ( Schouten *et al.*, 2020). However, the majority of substantive analyses collecting images have occurred outside the scope of web surveys.

Overall, there is limited empirical evidence. Moreover, the few existing studies asking for images differ from our study at several levels: they deal with different topics ( *i.e.,* they demand photos of different places/objects than inside the dwelling/books), have been implemented in different countries and populations which might present different willingness and participation rates when it comes to sharing visual data, have allowed participation also from computers which might also affect the type of participation, have been programmed using different tools, have asked for a determined number of images, among others. Because of these disparities, it becomes challenging to anticipate the outcomes of our study accurately. Further research is essential to investigate how these diverse factors impact participation, data quality, and respondents' evaluations, but also to compare results obtained in more conventional ways to results obtained through images shared in the frame of web surveys.

Finally, it is worth mentioning that gender is one of the control variables to be used during the analyses since previous research suggests that differences might exist across genders ( Iglesias & Revilla, 2023). Gender was also considered in the design stage as it was used as a quota to get a sample similar to the target population in Spain (see section Sample for more details).

### Study objectives

The main objective of this study is methodological: we want to study the feasibility of asking for photos of the books respondents have at home, and to investigate the quality of the data received, in comparison to the one obtained through conventional web survey questions.

In particular, our study starts filling several of the gaps existing in the current methodological literature by investigating the following dimensions:

- *Respondents’ preferences*: do respondents prefer to provide the information about the books in their dwelling through images or answering questions in conventional ways?
- *Participation*: what are the rates of participation, break-off and item non-response for image-based *versus* conventional answer formats?
- *Respondents’ evaluations of different ways of answering*: which is the evaluation of respondents when answering questions through different methods?
- *Compliance*: to what extent do respondents comply or not with the tasks proposed related to capturing and sharing images?
- *Data quality*: do images allow for higher data quality and/or additional insights compared to conventional response formats?

By answering these research questions, our study expands the existing evidence in several ways. First, we take steps toward a more objective and accurate assessment of the number of books in the household, which is a highly relevant characteristic in the social sciences. Second, our study delves into exploring how participants respond when asked about items within their dwelling, thereby paving the way for potential avenues of research concerning other household items. Third, we analyze quality beyond the pertinence of the images sent, actually contrasting the types of insights contained through image-based and conventional questions. Finally, we collect the visual data using a new tool ( *WebdataVisual*; see Revilla *et al.*, 2022), which could improve the overall user experience and decrease the break-off and non-response rates.

In addition to the methodological objectives, this study also has substantive objectives. Indeed, the survey has been designed in order to delve into different substantive dimensions, in particular:

- *Mechanisms explaining the relations between the number of books at home and students’ achievement:* is the relation between the number of books at home (measured with different methods) and students' achievement (as operationalized by school grades) mediated by the home literacy environment?
- *Persistence of effects of the number of books on students’ achievement:* to what extent do the effects of the number of books at home (measured with different methods) persist when considering child- and family-related characteristics ( *e.g.,* parental education, home ownership)?

## Protocol

### a. Requirements to participate in the survey

In order to participate in this study, participants had to fulfill some requirements, especially:

- *Provide informed consent*: participants were presented with an information sheet stating the specification of the study, including the topic, the data protection statements, the project fundings, and the methodologies to be used, as well as the fact that they might be asked to share photos and that in such a case they should not share personal data in these photos. If participants clicked “next”, they were then presented with a consent form: only those who provided their consent could continue with the survey.
- *Participate from a mobile device*: although most laptops and some desktops have a camera which could allow capturing photos of the books and sharing them during the survey, it would have been complicated to move around the dwelling with those devices in order to take the photos of interest. Thus, we decided to limit the participation to respondents using smartphones and tablets, which allow for capturing photos more easily. This decision was supported by previous studies conducted with the same opt-in panel in Spain ( Netquest), which have reported that smartphones are the primary device used by respondents to answer surveys ( *e.g.,* 69% in Iglesias & Revilla, 2023, or 64% among millennials in Bosch *et al.*, 2019b). In order to guarantee mobile-only participation, the survey was programmed such that if a participant tried to connect to the survey through a PC, the following message was shown: “This survey should be answered from a smartphone or tablet. Please access to the survey again from one of these devices”. Participants were blocked until they entered through a mobile device.
- *Answer from their main residence*: Iglesias and Revilla (2023) found that the most limiting factor for sending images is related to the availability of the data. In particular, when asking for photos that must be captured during the survey, they found that between 17.1% and 33.7% of the respondents are not in a situation allowing them to take such photos. In order to maximize the chances that respondents were in a situation allowing them to capture and send photos of the books they have at home through the survey, we opted for including the following message at the very beginning of the survey: “This survey should be answered from your main residence (meaning the place where you have most of your belongings). If you are not there right now, please come back once you are in your main residence.” However, we could not detect if participants were really at their main residence. Therefore, although the message was shown, we could not force respondents to comply with this requirement.
- *Be a part of the target population*: this survey was aimed to parents in Spain having at least one child living with them regularly who attended first, third, or fifth year of primary education (see subsection “Sample” below for more details). Thus, respondents who entered the survey were asked whether they had children in primary school, and the grade they attended, and only those fitting with our target population were allowed to continue. Finally, when quotas were full (see subsection “Sample”), participants whose characteristics matched those of such quotas were filtered out ( *i.e*., redirected toward another survey of Netquest, usually a profiling survey used by the fieldwork company to gather information about their panelists that they can later use for selecting the samples more efficiently).

### b. Experimental design

***Methods to be tested***

We tested two main methods of answering that will allow us comparing the quality of conventional and image-based response formats:

- *Text*: Respondents had to provide the information about the books they have at home ( *i.e.,* number, language, and storage) by answering 11 questions using conventional formats ( *i.e.,* radio button or textbox).
- *Images*: Respondents had to provide the information about the books they have at home by sending photos of all these books.

Further, we considered a third method ( *TextPlus*) for the number-of-books questions, which is similar to *Text* but adds illustrations of reference, so respondents can have an estimate of what a certain number of books looks like. A message stating the number of books and the length of the shelf containing them was also presented. This method was included because previous studies suggest that a potential way of getting more accurate answers could be to present either illustrations of reference (see Heppt *et al.*, 2022), or sentences explaining the ratio between the number of books and the length of a shelf (see Sieben & Lechner, 2019). Thus, it is important to investigate whether proposing to share photos can improve the assessment of the number of books at home not only compared to a simpler *Text* format but also compared to the potentially improved version ( *TextPlus*).

**Figure 1.**
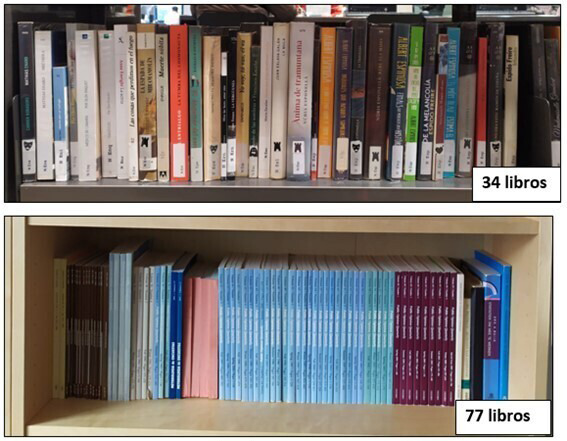
Illustrations of reference to be shown to those in group TextPlus. “libros” is the Spanish word for “books”. The use and reproduction of the images have been authorized by their author.

The illustrations of reference displayed in the survey are presented in Figure 1. These specific illustrations were designed by adapting the one used in Heppt *et al.* (2022), which showed a similar bookcase five times with different quantities of books, each of them matching the intervals in the response scale. We decided to use two shelves of the same length but with books of different thickness, to exemplify how the amount can vary depending on the types of books.

***Experimental groups***

Respondents were assigned to four groups, each being presented with different combinations of the three methods previously presented ( *Text*, *TextPlus*, *Images*):

- **Choice**: Respondents could choose which method they wanted to use for sharing the books' information: 11 conventional questions (subgroup called **TextChoice**) or capturing and sharing photos (subgroup called **ImageChoice**. Respondents could also answer “I do not have a preference”. In such a case, they were assigned to the **ImageChoice** subgroup. The method *TextPlus* was not offered.
- **Text-TextPlus**: Respondents were first presented the 11 conventional questions and asked to answer in conventional ways ( *Text*). Later, they were asked the four questions about the numbers of books a second time but including the illustrations of reference ( *TextPlus*).
- **TextPlus-Images**: Respondents were asked the 11 conventional questions first, and afterwards they were also asked to capture and share photos. The four conventional questions about the number of books displayed the illustrations presented in Figure 1 ( *TextPlus*).
- **Images-Text**: Respondents were first asked to capture and share photos of the books, and then they were asked the 11 conventional questions (without illustrations).

**Figure 2.**
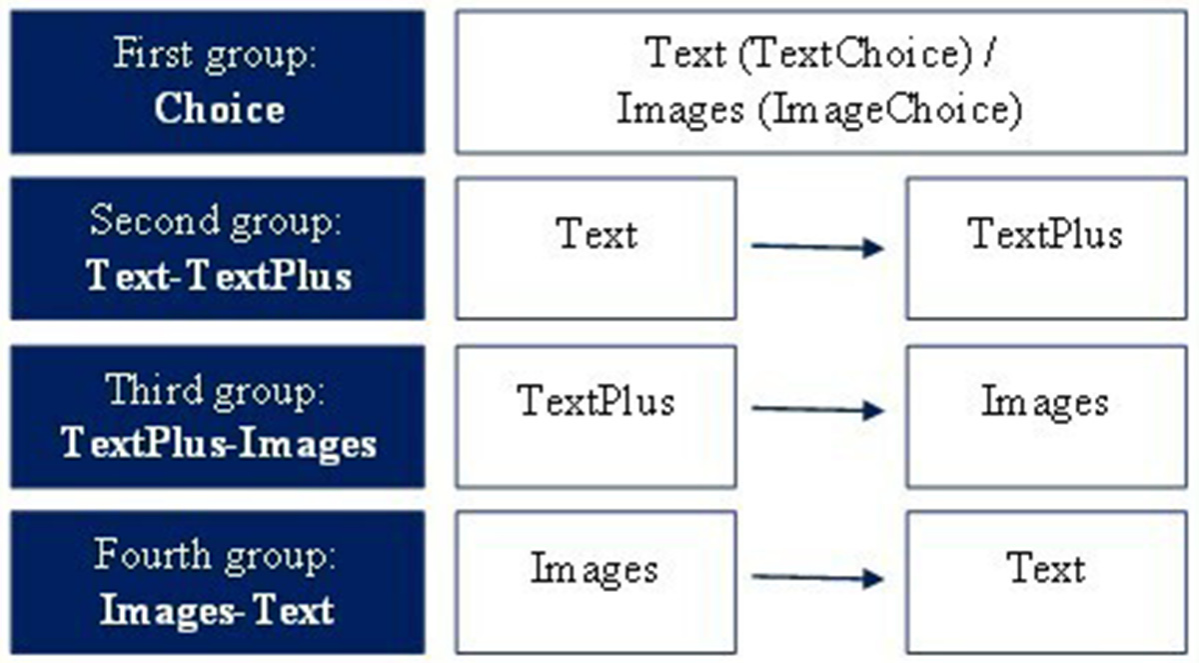
Scheme summarizing the experimental groups in the survey.

The scheme in Figure 2 summarizes the groups and subgroups of the experiment.

These groups were chosen because they allow fulfilling several goals. First, the group **Choice** was included in order to study the preferences of respondents.

Then, the other three groups have been chosen for the following reasons:

- The first moment of measurement allows comparing the three proposed methods ( *Text vs*. *TextPlus vs*. *Images*). The preferred method for the group **Choice** will also be part of such a comparison.
- Each group answered the questions regarding the books at home twice with different methods, so we can study whether specific combinations of methods and their order affect the results. Since it is not possible to know the true value, comparing the same method in two moments might help understanding some potential errors. For instance, we might identify that respondents tend to report higher numbers of books when they are asked conventional questions first (which could be due to social desirability). On the contrary, if they already sent photos of the books (so they know the researchers can see how many books they really have), they might be more careful about not over-reporting their number of books, leading to lower reports of the number of books.
- These groups should allow estimating the reliability and validity of the three methods of interest through the analyses of a three-group split-ballot MultiTrait-MultiMethod (MTMM) experiment ( Saris *et al.*, 2004). In such a design, three correlated traits (here, measured by the questions about the number of different types of books, see next subsection) are usually measured using three methods (here, *Text*, *TextPlus* and *Images*). If the model is identified in practice (which is not always the case, see Revilla & Saris, 2013), then estimates of measurement validity and reliability can be obtained for each trait and method. When using a three-group design where each method is answered once at time one and once at time two, differences depending on the position of the method can also be considered.

### c. Main experimental questions

The main experimental questions in this study are the ones about the books respondents have at home.

For the conventional questions, the questionnaire included four experimental questions regarding the number of books. For analytical purposes, we proposed different categories of books considering the estimated audience of such books based on age and literacy:

- The total number of books at home.
- The number of books for children who do not read by themselves.
- The number of books for literate children and teenagers.
- The number of books aimed to a general audience.

If respondents answered “Don’t know” for any of these categories, a follow-up question requesting for an approximate number of books in the respective categories was shown. In this follow-up question, respondents also had the opportunity to answer “I am unable to provide an approximate number either”.

In addition, other experimental questions were used to measure the following relevant aspects of the books:

- *The books’ language(s)*: Three questions were asked about the proportion of books in 1) Spanish, 2) the co-official languages in Spain (Catalan, Euskera and Galician), and 3) other languages. These questions, aimed to assess the extent of the use of different languages within the household, are particularly relevant for explaining the performance in Spanish class.
- *The places where the books are stored*: Four questions were used to assess whether respondents keep some of their books in 1) shelves, 2) center, coffee, or night tables, or over a desk, 3) inside closets or drawers, and 4) in a different place than the others mentioned. These questions allow getting an understanding on how visible and accessible the books are for the household members.

**Figure 3.**
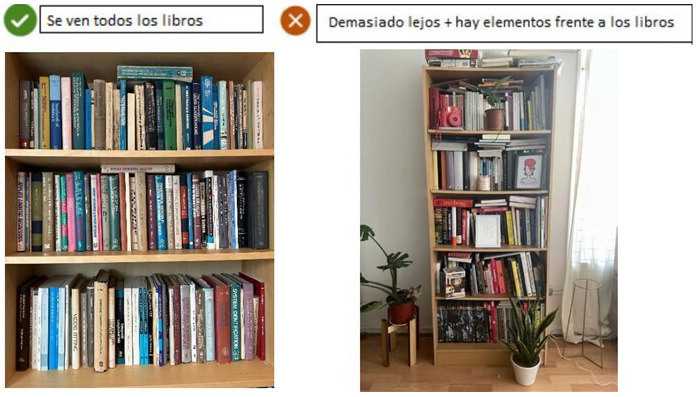
Examples of good and bad photos. The message “Se ven todos los libros” means “All books are visible”. The message “Demasiado lejos + hay elementos frente a los libros” means “Books are too far + they are covered by some items”. The use and reproduction of the images have been authorized by their authors.

For the image request, respondents were only asked one question, in which they were instructed to take and share photos of all the books in their main residence. Although it was only one question, it was longer than the conventional ones since it presented the instructions on how to take the photos, in particular:

- *Which items should not be captured* ( *e.g.,* schoolbooks, magazines and e-books). Schoolbooks and magazines are usually excluded in most studies asking for the number of books (see some examples in Brunello *et al.*, 2012; Engzell, 2021; Sieben & Lechner, 2019). We also excluded e-books for two mains reasons. First, previous research (e.g., Heppt *et al.*, 2022) has found that e-books do not have an influence on the children’s academic performance (contrary to printed books). Since this study focuses on these relations, including e-books does not seem necessary. Second, this could add an additional burden to respondents, and since we already asked them an unusual task, we preferred to avoid another special task in this first study.
- *Framing of the photos*: The instructions encompassed several key points to ensure effective book capture and readability in the photos. First, they stressed that it was essential to capture the full book, including the titles for easy identification. When taking multiple photos, respondents should strive to avoid duplication by ensuring that each book appeared in only one image. Additionally, any decorations covering the books must be removed before taking the photos. Lastly, in cases where books were located in different places, separate photos should be taken for each location and sent accordingly.
- *Remarks regarding personal data*, reminding respondents of not sending photos containing their own or third parties’ personal data.
- *Visual examples of good and bad photos*: Figure 3 provides a copy of these examples.
- *Characteristics of the tool allowing the photos:* For instance, the instructions stated that the photos can be deleted, where to press to capture the photo, and how to send more than one photo.

For the full instructions, see the translated version of the questionnaire (to English) available as extended data of the project (see the Data Availability section).

Based on these photos, we plan to extract the relevant information in order to get data regarding the same 11 aspects measured in the conventional questions. For instance, to classify the books into each of the three categories, we plan to use the color and fonts, but also shapes and any other useful indicator ( *e.g.,* editorial, titles, or authors). The classification will be done at least manually (two classifiers will be involved; with a small part of the images being classified by both), even if we are also considering the possibility of implementing automatic classification.

### d. Other experiment-related questions

Besides the main experimental questions, respondents were asked some additional experiment-related questions depending on the group to which they belonged, and the conditions associated to such group (see Table 1 for a summary of the questions the different groups had to answer):

**Table 1.** Experimental and experiment-related modules to be answered by each group.

Module	ImageChoice	TextChoice	Text-TextPlus	TextPlus-Images	Images-Text
First question about preference	Yes	Yes	No	No	No
Conventional questions regarding number of books **without** the illustrations of reference	No	Yes	Yes	No	Yes
Conventional questions regarding number of books **with** the illustrations of reference	No	No	Yes	Yes	No
Rest of conventional questions regarding books (language and storage)	No	Yes	Yes	Yes	Yes
Assessment of how the illustrations helped	No	No	Yes	Yes	No
Reasons for not choosing images	No	Yes	No	No	No
Easy and like of conventional questions	No	Yes	Yes	Yes	Yes
Reasons for dislike of conventional questions	No	Depends	Depends	Depends	Depends
Image-based question regarding books	Yes	No	No	Yes	Yes
Easy and like of image-based question	Yes	No	No	Yes	Yes
Reasons for dislike of image-based question	Depends	No	No	Depends	Depends
Reasons to not upload photos	Depends	No	No	Depends	Depends
Difficulties while submitting photos	Yes	No	No	Yes	Yes
Second question about preference	Yes	Yes	No	No	No

- **Choice**: respondents in this group were initially prompted to select their preferred method ( *Text* or *Images*) for answering questions about books. After providing their responses using their chosen method, they were subsequently asked to reconsider and indicate which method they would opt for if faced with the same choice again.
- **TextChoice**: respondents were asked their reason(s) for not having chosen to capture and share photos.
- **Text-TextPlus** and **TextPlus-Images** groups: respondents were asked to assess whether the illustrations of reference helped them in the estimation of the numbers of books.
- All except **ImageChoice**: respondents were asked how much they dis/liked and found easy/difficult answering the conventional questions (with or without the illustrations), and the reasons for those stating they did not like answering them.
- **ImageChoice**, **TextPlus-Images** and **Images-Text**: respondents were asked how much they dis/liked and found easy/difficult answering by sharing images, and the reasons for those stating they did not like answering in this way.
- **ImageChoice**, **TextPlus-Images** and **Images-Text**: respondents were asked for their reasons for not uploading photos (if this was the case) or difficulties while uploading the photos (to those who uploaded at least one image).

### e. Full questionnaire

Overall, the questionnaire used in this study included up to 65 questions (for a full draft of the questionnaire in Spanish and English, see the “Data availability” section of this protocol). However, due to routing and to our experimental design, on average, each respondent was asked around 55 questions.

In addition to the main experimental questions (presented in section c) and experiment-related questions (presented in section d), the questionnaire covered the following dimensions:

- Sociodemographic characteristics of respondents, including age, gender, and educational level. These variables were needed for the quotas, and/or will be used as control when performing the regression analyses (see subsection h “Analysis plan”).
- Number of children and characteristics of (one of) their children in primary school (child-related characteristics such as year of birth, gender, language spoken at home; school-related characteristics such as year of primary education, grades in Spanish and mathematics, type of school attended). These will allow characterizing the participants. Furthermore, most of them will be included in the substantive analyses.
- Activities related to the children’s and/or family’s involvement with literature and questions on the family’s housing situation. These questions were included to fulfill the substantive goals of the study.
- Usage of camera-related functions with their mobile devices and comfort with new technologies. These questions were included to measure the level of familiarity that respondents have with technologies regarding the capture and sharing of photos, and investigate whether it affects, for instance, the level of participation or preferences.
- Self-assessment of their spatial, mathematical, and verbal abilities. This set of questions will be used to identify whether these factors influence the accuracy of the answers.

The questionnaire was programmed using mainly a paging design; however, up to four questions were in some cases presented together in one page. Questions were not mandatory ( *i.e.,* respondents could continue the survey without answering them), except for those used for quotas, filters, and those that conditioned the wording of the following questions. However, a pop-up warning message was shown if a fourth question was left without an answer, in order to motivate participants to provide responses. Warning messages were also displayed if one or more questions remained unanswered when several questions were presented on the same page. Interruptions were allowed (i.e., respondents could leave and resume the survey later). Respondents were not allowed to go back to previous questions. Gender neutral language was used in most questions. However, it was not used in two cases: first, when a question ended up too long or complicated to read when using gender-neutral language. In those cases, the questions were personalized according to the self-declared gender of the participants (based on the question “Which gender do you identify with?”. The options “Male” and “Female” were offered). Second, when asking questions about one of the respondents’ children, questions were personalized with the child’s gender to both keep the questions shorter (in Spanish, there is not a gender-neutral word for “child”) and minimize the risk of respondents thinking about another of their child than the one they were answering about (for parents of children of different gender).

### f. Sample

Our target population included all adults (18+) living in Spain who had at least one child in the first, third or fifth year of primary school living with them regularly. The target population was chosen because, as presented in the objectives, the data will also be used to assess the relations between the number of books at home and the grades obtained in school by children in primary education. Although primary school in Spain encompasses Grades one to six, we decided to focus only on three cohorts due to changes in the evaluation system in the country that were only applied to Grades one, three, and five during the 2022–2023 academic year ^i^: students would receive, instead of numerical grades, qualitative assessments of their performance. Thus, including all the cohorts might have produced a loss of data quality since children would have different evaluation systems and scales.

Data collection was implemented in June 2023 in the Netquest online opt-in panel in Spain ( http://www.netquest.com), which offers rewards ( *i.e.,* points that can be redeemed for gifts) to the respondents for each completed survey.

Quotas for age, gender, and educational level of respondents were used to get a sample that, on these variables, closely resembled the population of adults with children between six and 12 years (i.e., the average ages of children attending primary school in Spain) in the Economical Active Population Survey conducted by the National Statistics Office of Spain ^ii^.

The crossed-quotas for gender (self-reported after being asked to choose between “male” and “female”) and age were: 13% for males between 18–39 years old, 23% for females the same age, 35% for males aged 40 or more, and 29% for females the same age. As for education, 55% were assigned to those up to secondary, and 45% for those with some type of tertiary education. We used a margin of +/- 3 percentage points for each group since, even if we used official data to calculate quotas, our target population is not exactly the same as the one in these official statistics. Thus, we decided to allow the distributions of the groups to vary to some extent.

From 4,854 invited individuals, a total of 2,443 started the survey. Out of them, 899 were filtered out due to non-compliance with security checks or survey requirements (including 151 individuals who did not consent to participate). 72 individuals were excluded as their demographic profile had already met the required quota. 270 started the survey but later broke-off. Thus, 1,202 individuals completed the survey until the end (25% of those invited). Within the group who finished the survey, 52% of participants were female, the mean age was 42 years, and 45% counted with a higher education degree.

Since respondents were assigned to one of the four experimental groups considering the group with the smallest number of participants at the moment the respondent was allocated ( *i.e.,* just before starting the experimental block), all groups have very similar sizes (300 or 301 participants).

### g. Tool to collect the images

**Figure 4.**
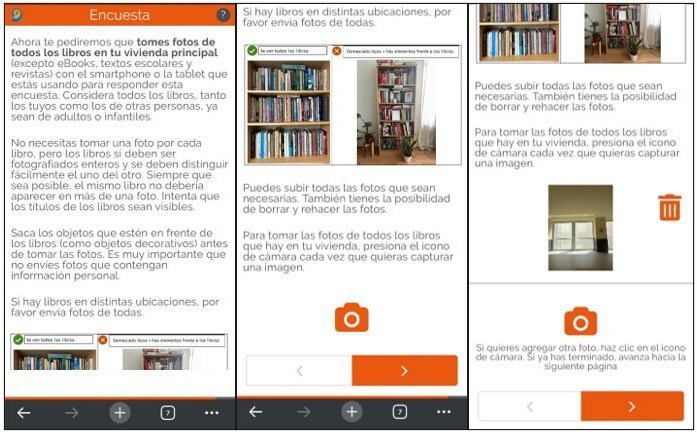
Screenshots of the tool *WebdataVisual* in the survey (including the capture and delete buttons). The use and reproduction of the images has been authorized by the programmer and by the person doing the screenshots.

The image collection in the survey utilized the *WebdataVisual* tool ( Revilla *et al.*, 2022), which enables both capturing and sharing photos during the survey, and submitting already stored images. In this study, only the function to take and submit photos captured during the survey was used. The tool allows taking, previewing, and eventually deleting and retaking photos. Some screenshots of the book question programmed using this tool are available in Figure 4.

*WebdataVisual* offers various data types related to the captured photos. In this study, we collected the following information:

- File format: each file’s format is stored in a string variable with the name of the format.
- Number of uploads: this displays the total count of photos uploaded by each respondent, irrespective of whether they were deleted or not.
- Number of deletions: it indicates the quantity of images uploaded and subsequently deleted by respondents.
- Number of server uploads: this represents the number of photos uploaded to the server, excluding the deleted ones.

*WebdataVisual* does not collect metadata like the location where the photo was captured to avoid unnecessary collection of personal information.

### h. Analysis plan

***Image classification***

Before conducting the actual analyses, there is a preliminary step involved in the image collection process, known as image classification. This step entails extracting relevant information from the images and converting it into labels/codes for subsequent analysis ( Gavali & Banu, 2019), similar to other survey-produced data. To classify the images, we will follow the steps proposed by Iglesias, Ochoa and Revilla (2023).

Initially, our coding framework for the images includes the following aspects:

- Visual clarity and analyzability identification: assessing the general visual clarity of the images and its potential for analysis.
- In line: assessing whether the content of the images is in line with what was asked.
- Overlap: noting instances where the same book(s) appear in different photos from the same respondents. In such cases, the book(s) should only be counted once ( *i.e.,* in the first image).
- Counting identification: verifying if the number of books in the images can be identified.
- Book category identification: determining whether there is enough information in the images to classify the books into the categories of interest ( *i.e.,* books for children who do not read by themselves, books for literate children and teenagers, and books aimed at a general audience).
- Number of books per category, per image: recording the count of books per category for each image, and the count of books that cannot be placed into a category.
- Storage identification: verifying whether the storage of the books can be identified.
- Storage type: defining the type of storage.
- Title identification: evaluating whether the titles of the books in the images can be read.
- Language: stating whether there are books in Spanish, in another of the co-official languages in Spain, and in other languages.

This initial coding framework may be subject to change after inspecting the photos and discovering new or unexpected information during the analysis process.

Two human classifiers will classify the photos, following the guidelines created for the project. The tentative classification guidelines are available as extended data of the project (see the "Data availability" section). The classifiers will have a percentage of images in common in order to compare results. In a future, we will also consider whether automatic coding might be used.

Once the image classification will be completed, we will proceed with the actual analyses, which will be categorized into two main blocks: methodological analyses and substantive analyses. In both of these analyses, the variable identifying the respondent’ and/or the child’s gender will be used as control, since it is expected that it might affect the results.

**Methodological analyses**

The methodological analyses will encompass various aspects, including preferences, participation, compliance with tasks, data quality, and respondents' evaluations.

Descriptive analyses will be conducted to compare proportions of respondents' preferences, rates of participation, and overall evaluations of different ways of answering ( *e.g.,* responding with or without illustrations) between conventional questions and image-based formats. We will also test for the significance of the differences across groups and/or formats.

Furthermore, regression analyses will be performed to examine whether and to what extent different aspects of interest (preferences, participation, evaluation, compliance, and data quality) are influenced by respondents' characteristics, such as gender, age, level of education, frequency of using the device's camera, and prior experience as a Netquest panelist.

By conducting these methodological analyses, we aim to gain insights into the effectiveness and efficiency of utilizing image-based formats in comparison to conventional methods, as well as the potential impact of respondents' characteristics on their responses.

Regarding data quality, different indicators will be considered, such as non-valid answers, amount of relevant information that can be obtained, or predictive validity. Moreover, we plan to use structural equation modeling to estimate a true-score MTMM model ( Saris & Andrews, 2004) with the number of books in the different categories as traits and *Text*, *TextPlus* and *Images* as methods. If the model is identified in practice, this would enable to estimate the validity and reliability of these traits when using conventional versus image-based methods to obtain the information and see which one performs better.

**Substantive analyses**

To investigate the mechanisms explaining how the number of books relates to academic achievement (measured by the children's evaluations in Spanish and mathematics), and whether the effects of books-at-home on children’s academic achievement remain statistically significant when other variables (such as parental education, home ownership, or length of living at the same dwelling) are controlled for, structural equation modeling will be performed. These analyses will be performed for the number of books collected through conventional and image-based formats.

Further, exploratory analyses regarding academic achievement for students with mostly books in a minority language might be performed, especially when analyzing the grades in Spanish (the majority language in Spain).

### i. Plans for dissemination

We expect to write at least three scientific papers with the data collected through this survey: one regarding the participation, preferences, and evaluations of the experimental questions, another regarding data quality, and at least one studying the substantive questions presented in the objectives.

Those papers will be presented in national and international conferences, and their findings will be used to teach short courses regarding the use of new data types in web surveys. We also plan to develop webinars and disseminate the results in social media.

The anonymized dataset together with all the documentation about the project and scripts used to do the analyses will be made publicly available in the OSF folder of the project once the study is completed and the main results have been accepted for publication. The images will not be published, but only the information (codes) extracted from these images.

### j. Ethical and data protection issues

When collecting images, it is crucial to consider data protection and ethical aspects. The primary concern pertains to participants (inadvertently) sharing personal data of themselves or third parties. To address this, respondents were explicitly instructed to not include photos with personal data already in the information sheet and then again in the experimental question asking for photos. However, despite their agreement not to share personal data, some respondents might (possibly unknowingly) send images containing such information.

To mitigate this risk, all collected images underwent a two-step manual review by the online fieldwork company (Netquest) and the project’s Ethics Advisor. 42 participants sent photos with personal data, most of which corresponded to family photos in front of/next to the books. Any images containing personal data were identified, and the Ethics Advisor obscured the data to ensure it was not visible during the classification stage. Protocols were established for unexpected findings involving legal obligations ( *e.g.,* domestic violence), but fortunately, it was not necessary to implement such measures. The revised visual files were then shared with the research team.

The classification of information within the visual files will be conducted manually by one or two of the team members. There is also a possibility of utilizing a machine learning algorithm, which could be implemented by a team member or an external collaborator, following the signing of a non-disclosure agreement.

Due to the sensitive nature of these data, the images collected through the survey will not be shared in the OSF repository for external use. However, for the sake of transparency and reproducibility, both the full guidelines used to classify the images and the codes extracted from the images will be made available.

Finally, the WEB DATA OPP project, of which this study is part, has received ethical approval from the Institutional Committee for Ethical Review of Projects (CIREP) in the Universitat Pompeu Fabra (17.12.2019, CIREP- approval no. 135) and confirmation from the Data Protection Officer of the Universitat Pompeu Fabra (20.12.2019). This approval ensures that the project adheres to ethical guidelines and safeguards the rights and privacy of participants. Further, this particular study was approved by the project’s Ethics Advisor.

## Consent

Written informed consent for participation in the project and publication of results was asked to survey respondents prior to participating in the survey. The form is available as extended data of the project (see the "Data availability" section).

## Conclusions/ discussion

This document presented the protocol for a study collecting information regarding the books people have at home through conventional and/or image-based response formats. Depending on the group respondents were assigned to, they had to a) answer 11 conventional questions regarding the number of books they have at home (with or without illustrations), the books’ language(s), and the way they are stored, and/or b) send image(s) of such books. Moreover, respondents received some questions to assess their overall evaluations with different ways of providing the information, and others to gather the data needed for the substantive analyses.

We anticipate that this study will contribute significantly to the expanding body of literature on collecting new types of data through web surveys. However, this study presents some limitations. First, the study is based on an opt-in online panel, which might introduce biases in the sample, as participants self-select to be part of the panel. Nevertheless, plenty of online surveys nowadays are done through such panels ( García Trejo *et al.*, 2022), so it is relevant to study them.

Second, the focus on a specific target population, namely parents of children in first, third, or fifth year of primary school in Spain, might lead to limited generalizability of the findings. This group could possess unique characteristics, such as time constraints due to childcare responsibilities, which could impact their ability to capture photos throughout their homes. Thus, it is not appropriate to extrapolate the study’s conclusions to other panels (whether opt-in or not) or different populations.

Third, since the true values are unknown (e.g., we will not be able to go to the houses to check the actual number of books and their languages or storage), there exists inherent uncertainty about the accuracy of the results. For instance, we cannot be sure that the images collected cover all the books present in respondents’ homes. However, by comparing the answers provided in conventional ways and by sharing images, we will get some insights about possible issues of this kind. Moreover, this uncertainty applies to virtually all prior studies relying on conventional methods for assessing the number of books at home.

Finally, there is no assurance that respondents were at home when answering the survey, which might influence their non-response to the image-based question.

Even if researchers should interpret the findings within the context of the aforementioned constraints to ensure a nuanced understanding of the results, the study provides valuable insights into the collection of visual data through web surveys and serves as a stepping stone for future research in this area. Moreover, by conducting both methodological and substantive analyses, we will achieve a comprehensive understanding of the data about the books respondents have at home collected in different ways and its potential practical implications for further research.

## Data Availability

Open Science Framework (OSF): A survey experiment about the books in the respondents’ main residence. https://doi.org/10.17605/OSF.IO/KHTEP This registration contains the following extended data: Data file 1: Questionnaire in English including the information sheet and informed consent. Data file 2: Questionnaire in Spanish including the information sheet and informed consent. Data file 3: Tentative classification guidelines. Data file 4: Screenshots of the programmed survey. Data will be available under the terms of the Creative Commons Attribution 4.0 International license (CC-BY 4.0).

## Footnotes

^i^ See https://eurydice.eacea.ec.europa.eu/national-education-systems/spain/national-reforms-school-education

^ii^ The public dataset (available at https://www.ine.es/dyngs/INEbase/es/operacion.htm?c=Estadistica_C&cid=1254736176918&menu=resultados&idp=1254735976595#!tabs-1254736030639) displays the age of the members of the dwellings in ranges of 5 years. Thus, for the estimation aimed to calculate the quotas, dwellings with children between 5–9 years old during the first trimester of 2022 were considered, since those children will be 6–10 years old in the same trimester of 2023. Thus, our quotas are a proxy for dwellings with children between 6 and 12 years old, since it is not possible to know that exact range of ages from the publicly available data.

## Software availability

The programming of the survey was performed by Netquest. The company used their own software, which is not openly available. Although there are platforms allowing to conduct surveys for free (such as SurveyMonkey or Qualtrics), their free versions are not compatible with our study due to the programming needed for collecting the images, which is usually not included in their cost-free versions.

The collection of the images was performed by using the *WebdataVisual* tool ( Revilla *et al.*, 2022). Although the code of the tool is openly available, it is necessary to integrate it with a survey software, which in our case was the one of Netquest as mentioned previously. Moreover, the images collected through *WebdataVisual* need to be stored in a server (e.g., we used AWS), which is not available for free.
