# Supplementary material for: Protocol for a web survey experiment studying the feasibility of asking respondents to capture and submit photos of the books they have at home and the resulting data quality
Source: Open Res Eur. 2023 Nov 20;3:202. [Version 1] doi: 10.12688/openreseurope.16507.1 (PMC11019288; doi:10.12688/openreseurope.16507.1)
Supplement: Peer review history

# Reviewer report: Reviewer response for version 1

**DOI:** 10.21956/openreseurope.17821.r36816

**Published:** 2024-04-15

**Authors:** Berislav Žmuk

**Affiliations:** 1 University of Zagreb, Zagreb, Croatia

The protocol for a web survey related to studying the feasibility of asking respondents to capture and submit photos of the books they have at home, and the resulting data quality, is very detailed and describes the process in great depth. However, there are some points in the protocol that require additional thought and improvement.

 Firstly, the mobile web survey was planned and conducted. According to the screenshots of the survey, it can be noticed that in some cases, there can be a lot of small text on the display. Also, some questions require scrolling on the screen, which can be exhausting for respondents; they could overlook a question, etc. Therefore, it is recommended to go through the questionnaire design again and make it more user-friendly on mobile devices.

 You are limited to a population that has smartphones or tablets. What about the population that does not have smartphones or tablets? Is that population statistically significantly different? Should this population be covered in a different way? Also, what if a respondent has a smartphone but limited access to the Internet? Are respondents who do not want to take photos due to privacy concerns statistically significantly different from other respondents? These practical and technical limitations should be addressed more thoroughly.

 The target population should be better elaborated. Are you covering just parents or all adults (in both cases, +18)? Is it possible that an 18-year-old person reports for his little brother? If you are observing parents 18+, is it possible that an 18-year-old mother has a child in the first, third, or fifth year of primary school? What if there are more children in the household—one in the first, the second in the third, and the third in the fifth year of primary school? Did you take into account such possibilities? Does it have any relevant impact on the results? What does it mean for a "child to be living with them regularly"? You should be more precise and concise.

 Do you plan to conduct a survey about the number of books without asking respondents to take photos of them? It would be interesting to compare those two groups and check whether there is some over or underreporting tendency.

 Are you collecting any additional paradata (location, survey time, location of breakoffs, etc.), and do you plan to use them in the analysis?

 Methodological analyses are not so detailed. For example, it is not known which statistical tests you will use to test for the significance of the differences across groups and/or formats or which kind of regression model you will apply. Do you plan to conduct the analysis on the whole sample level, or do you plan to do it, for example, for females and males, regions, etc.?

 Do you think that AI would be reliable enough to go through the pictures and collect data about the number of books, titles, etc.? That would be a great finding. In that way, maybe more respondents would participate in the survey because it would be easier to take a photo of something than to describe it. Perhaps another paper could be written in that direction.

Is the study design appropriate for the research question?

Yes

Are sufficient details of the methods provided to allow replication by others?

Yes

Is the rationale for, and objectives of, the study clearly described?

Yes

Are the datasets clearly presented in a useable and accessible format?

Yes

Reviewer Expertise:

economics, business statistics, survey methodology, statistical quality control

I confirm that I have read this submission and believe that I have an appropriate level of expertise to confirm that it is of an acceptable scientific standard.

# Reviewer report: Reviewer response for version 1

**DOI:** 10.21956/openreseurope.17821.r36564

**Published:** 2024-03-28

**Authors:** Ioannis Andreadis

**Affiliations:** 1 Aristotle University of Thessaloniki, Thessaloniki, Greece

This protocol presents a very interesting research design regarding the collection of images through web surveys, focusing on data about the number of books that the survey participants own. This is a very well written protocol and all the questions that have occurred to me while reading the text, have been answered later in the document.

 For instance, my first question occurred early in the introduction, and it was about the exclusion of virtual or e-books, a decision that at that point of the test was presented without any convincing arguments. However, later in the text, it was made clear that the decision was made for two reasons: i) according to the literature, e-books do not have an impact on children’s academic performance, and ii) given that the main methodological task is on the performance of the submission of photos through a web survey, excluding e-books will reduce respondents’ burden without affecting the main methodological task. At this point, I would like to note that I am fully covered with the justification of the second reason, but if the authors would like to maintain the first reason, I think that one study only is probably not adequate and I would suggest them to provide additional publications that confirm the findings of Heppt et al.

 On another issue, the authors mention that sharing images takes more time for participants. I would like to see more details about this finding. Is it based on the overall duration of the survey, or on the time spent to answer the questions about the books only? Does it depend on the number of the books or uploaded photos? In addition, if the authors want to compare times, it will be useful to compare with varying levels of information asked from the respondents. For instance, the comparison would probably give different outcomes if the “Text” group was asked to provide all the information about their books that is available through the photos. In fact, this would also have an impact on the distribution between text-choice and image-choice (e.g. who would choose the text option if the respondents were asked to choose between i) typing the titles of all their books and ii) uploading their photos?

 Finally, I would like to make a suggestion regarding the third limitation mentioned by the authors: although we do not know if the photos cover all the books owned by the respondents, we know that this is the lower bound of this value (i.e. the actual number of the books cannot be smaller than the number of the books on the photos). Consequently, this lower bound can be used to identify respondents who have provided a text response that underestimates the actual number of the books they own.

Is the study design appropriate for the research question?

Yes

Are sufficient details of the methods provided to allow replication by others?

Yes

Is the rationale for, and objectives of, the study clearly described?

Yes

Are the datasets clearly presented in a useable and accessible format?

Yes

Reviewer Expertise:

web surveys, survey methodology, data quality, political representation, populist attitudes

I confirm that I have read this submission and believe that I have an appropriate level of expertise to confirm that it is of an acceptable scientific standard.

# Reviewer report: Reviewer response for version 1

**DOI:** 10.21956/openreseurope.17821.r37966

**Published:** 2024-03-23

**Authors:** Tarek Al Baghal

**Affiliations:** 1 University of Essex, Colchester, England, UK

Thank you for the opportunity to review this article describing the design for the survey study on books in the household. Various methods are compared; the crucial comparison is between conventional questions and requesting that respondents take and upload photos of books in the household. The data collected from this study will first compare the relative merits of the methods employed and then to identify how these data predict substantive outcomes. The nature of this study is important given that technologies allow for alternative data collection and possibly improve measurement. 

 There are a few concerns with the design which could be addressed with additional discussion, and possibly with revision of statistical methods. 

 One issue is the sample sizes for the design. The n is 1202; is 1/2 are asked to provide images (combining all ordering of requests), and on the high end they get 1/2 providing photos, that is an of 300 with images. They then want to try to extract an order effect, and given the choice category, only 100 in the second placement. Given that the order is not fully crossed, extracting ordering effects are based on certain assumptions of the model. Are these sample sizes adequate for identifying everything they hope to? On this point, was there anything done to ensure higher participation rates? If not, and the percent providing photos is toward the lower end, sample sizes would increasingly be problematic. 

 A second issue is the defined population, which is not a problem, and the traits being used for methodological tests. There are three types of books identified as measures/traits, for children needing assistance with reading, books for literature children and teenagers, and books for the general population. There is a reasonable expectation respondents would not have books in some of these due to the population. Given the population includes families with children in grades 1, 3, and 5, there is no reason for any of these families to have books for literature teenagers, except by chance, not by design. 

 Similarly, while it may be that there will be books for children and the general population, whether there are books for those needing assistance or for literature readers would be dependent on the age of the child. For example, if a family had a child in year 1 with no other children, having no books for literate children and teenagers makes sense, and is not an indicator of anything (possibly) other than the age of their child. That is, there are going to be structural zeros in the data that can't be compared to actual zeros. 

 These issues may be more of an issue in the MTMM method proposed. How much does small sample sizes affect the ability to extract shared variance from multiple indicators? How are traits that would not necessarily be had for a certain population treated? And how do these "weaker" traits affect the confidence in this methodology?

 Somewhat related is the issue if the classification of books if a photo is provided. I appreciate quality and compliance might be best judged by coders, but any poor photos will decrease the sample size for the traits component of the MTMM. While total books may be more easily identified, if title (in particular) is not available, how books are classified into the three traits is unclear. 

 However, there is clear merit to the study. The need for new and better ways to leverage technology in surveys to collect more and better indicators is certainly a worthwhile objective. The design and data from this study will surely be informative to these goals. Addressing the above issues will improve the confidence that the outputs (which are identified and appropriate) move understanding forward to the full extent these data are able.

Is the study design appropriate for the research question?

Partly

Are sufficient details of the methods provided to allow replication by others?

Partly

Is the rationale for, and objectives of, the study clearly described?

Yes

Are the datasets clearly presented in a useable and accessible format?

Yes

Reviewer Expertise:

Survey methodology

I confirm that I have read this submission and believe that I have an appropriate level of expertise to confirm that it is of an acceptable scientific standard, however I have significant reservations, as outlined above.
